# Supplementary material for: Modulatory Impact of Adipose-Derived Mesenchymal Stem Cells of Ankylosing Spondylitis Patients on T Helper Cell Differentiation
Source: Cells. 2021 Jan 30;10(2):280. doi: 10.3390/cells10020280 (PMC7912699; doi:10.3390/cells10020280)
Supplement: Supplementary file 1 [file cells-10-00280-s001.pdf]

Table S1. Comparison of demographic and clinical characteristics of male (M) and female (F) patients.

Parameters	Ankylosing spondylitis (M) (*n* = 12)	Ankylosing spondylitis (F) (*n* = 9)	P value
Demographics			
Age, years	44.7 ± 3.66	40.9 ± 4.49	0.536
Disease duration, years	8.83 ± 1.35	5.56 ± 1.13	0.09
Clinical data			
BASDAI, score	5.22 ± 0.62	6.11 ± 0.78	0.171
$ASDAS_{CRP}$, score	3.28 ± 0.29	3.63 ± 0.34	0.353
BASFI, score	4.49 ± 0.71	5.05 ± 1.14	0.847
BASMI, score	4.13 ± 0.59	3.78 ± 0.64	0.846
HAQ, score	1.02 ± 0.16	1.21 ± 0.37	0.785
Laboratory values			
CRP, mg/L	10.67 ± 3.64	17.13 ± 6.53	0,223
ESR, mm/h	14.58 ± 4.56	30.1 ± 6.8	**0.03**
Medications, % (n)			
NSAIDs	91.6 (11)	88.9 (8)	
Non-biologic DMARDs	16.6 (2)	33.3 (3)	
Glucocorticosteroids	8.3 (1)	22.2 (2)	

Explanations as in table 1.

Table S2. Comparison of cytokine production and generation of Treg cells in the co-cultures of target cells with ASCs of male (M) and female (F) patients.

Cytokine /cells	Sex (n)	Co-culture of $CD4^+$ T cells with		Cytokine / cells	Sex (n)	Co-culture of PBMCs with	
		ASCs	$ASCs_{STI}$			ASCs	$ASCs_{STI}$
**IFN**	M (4)	2512 ± 108	2516 ± 209	**IFN**	M (7)	378 ± 211	226 ± 114
	F (4)	2414 ± 98	2479 ± 56		F (4)	716 ± 710	239 ± 235
	P value	*0.625*	*0.886*		*P value*	*0.699*	*0.699*
**IL-17AF**	M (5)	389 ± 154	376 ± 168	**IL-17AF**	M (10)	444 ± 85	418 ± 85
	F (6)	1002 ± 329	1043 ± 288		F (5)	608 ± 137	501 ± 101
	P value	*0.143*	*0.143*		*P value*	*0.309*	*0.594*
**IL-10**	M (6)	2122 ± 859	1809 ±780	**IL-10**	M (14)	301 ± 40	355 ± 45
	F (6)	2364 ± 628	1890 ± 560		F (4)	367 ± 85	356 ± 82
	P value	*0.818*	*0.937*		*P value*	*0.367*	*0.958*
**TGF**	M (6)	112 ± 39	94.8 ± 30	**TGF**	M (7)	341 ±102	313 ± 181
	F (6)	413 ± 211	514 ± 182		F (4)	696 ± 244	441 ± 53
	P value	*0.798*	*0.328*		*P value*	*0.527*	*0.230*
**Treg cells**	M (6)	19.7 ± 3.4	18.4 ± 4.0	**Treg cells**	M (14)	10.0 ± 0.94	8.7 ± 0.98
	F (10)	13.3 ± 1.5	13.2 ± 1.0		F (4)	9.3 ± 1.7	8.1 ± 1.8
	P value	*0.073*	*0.492*		*P value*	*0.958*	*0.873*

Explanations: Data are expressed as the mean ± SEM; n – number of patients; P value for comparison of male vs female patients' ASCs.
